# Management of Type III Intrathyroidal Parathyroid Adenomas By Enucleation: Case Report and Literature Review

**DOI:** 10.7759/cureus.74167

**Published:** 2024-11-21

**Authors:** Ahmed M Youssef, Venkata Katreddy, Yasin Ahmed, Isabelle Nibelle, Arturo Mario Poletti

**Affiliations:** 1 College of Medicine, University of Sharjah, Sharjah, ARE; 2 Endocrinology, American Hospital, Dubai, ARE; 3 Pathology, American Hospital, Dubai, ARE; 4 ENT, American Hospital, Dubai, ARE

**Keywords:** blind surgery, hyperparathyroidism, intrathyroidal parathyroid adenoma, parathyroid enucleation, thyroidectomy

## Abstract

Parathyroid adenoma is a common endocrine disorder, but its intrathyroid presentation is relatively rare. The traditional approach, such as thyroid blind lobectomy, is the most frequent modality of treatment due to the possible unclear localization of the adenoma in the preoperative workup. This increases the risk of unnecessary probability of hypothyroidism.

We report a case of a 48-year-old male patient who was referred to the endocrinology outpatient clinic due to elevated calcium (Ca) and elevated parathyroid hormone (PTH) levels, indicating primary hyperparathyroidism (PHPT). Thyroid ultrasound (US) confirmed the presence of thyroid lobe nodule and the fine needle aspiration cytology (FNAC) revealed the presence of parathyroid cells. A thyroid scan with the single photon emission computed tomography (SPECT)/CT revealed a possible left inferior parathyroid adenoma. The patient underwent surgical excision of the left parathyroid adenoma by cervicotomy and thyroid lobe preservation with prompt normal PTH level recovery without complications. One year follow-up revealed no complications, and the patient's serum thyroid function, PTH, and Ca levels remained within the normal range.

The management of type III intrathyroidal parathyroid adenomas (iT-PAs) by enucleation offers a viable alternative for selected PHPT patients. Advanced diagnostic imaging helps but is often not able to confirm exactly their location. Individualized surgical approaches contribute to successful outcomes while preserving thyroid tissue. The establishment of standardized guidelines is essential to optimize the management of iT-PAs and enhance patient care. We present a case of a type III iT-PA by enucleation preserving the thyroid tissues along with the analysis of the few cases previously reported.

## Introduction

Primary hyperparathyroidism (PHPT) is a common endocrine disorder of 0.1-0.7% of the general population caused by excess parathyroid hormone (PTH) production [1]. Parathyroidectomy is the only effective and definitive treatment choice for the disease. The majority of PHPT was caused by parathyroid adenoma (85%). The incidence of intrathyroidal parathyroid adenomas (iT-PAs) has been reported between 1.3% to 6.7%, with an incidence of true intraparenchymal iT-PA is rare occurring in less than 1% of cases [2].

The ectopic position of missing abnormal parathyroid glands at cervical explorations is well-known and had been previously taught to lie in an intrathyroidal location [3]. The presence of parathyroid glands within the thyroid can be explained by their embryological development, while the inferior parathyroid originates from the third pharyngeal pouch, the superior parathyroid derives from the fourth pouch like the parafollicular C-cells of the thyroid. During the development at seven weeks the parathyroid glands separate from the pharyngeal wall and attach to the posterior surface of the thyroid [4]. Any incomplete separation or partial migration can result in an ectopic gland. Goodmans et al. classified the iT-PA into 3 classes: type I, the adenoma is under the thin connective tissue of the thyroid capsule but not within the thyroidal parenchyma; type II, the adenoma is partially within the thyroid tissue; type III, the adenoma is completely intrathyroidal [3].

Generally, the treatment of iT-PAs which is thyroid lobectomy indiscriminately increases the risk of postoperative hypothyroidism which is reported to occur in 14.3 to 35% [5]. We present a unique case that was classified as type III iT-PA. It had been locally enucleated avoiding the thyroid lobectomy.

## Case presentation

A 48-year-old male patient was referred to the outpatient endocrinology clinic in March 2022 to evaluate laboratory results showing elevated calcium (Ca) and elevated PTH indicating PHPT. He had a recent right 4 mm episode of nephrolithiasis with no bone complications. The results of laboratory examinations were compatible with PHPT (Table 1).

**Table 1:** Blood test results of the patient at first presentation PTH: Parathyroid hormone; Ca: Calcium; TSH: Thyroid-stimulating hormone

Blood work	Patient’s test results	Reference ranges
PTH	11.02 pmol/L	1.59-6.8 pmol/L
Serum Ca	2.53 mmol/L	2.1-2.5 mmol/L
TSH	1.2 mU/L	0.5-5.0 mU/L
Free-T3	4.0 pmol/L	3.1-6.0 pmol/L
Free-T4	14.3 pmol/L	11.0-21.0 pmol/L

**Figure 1:**
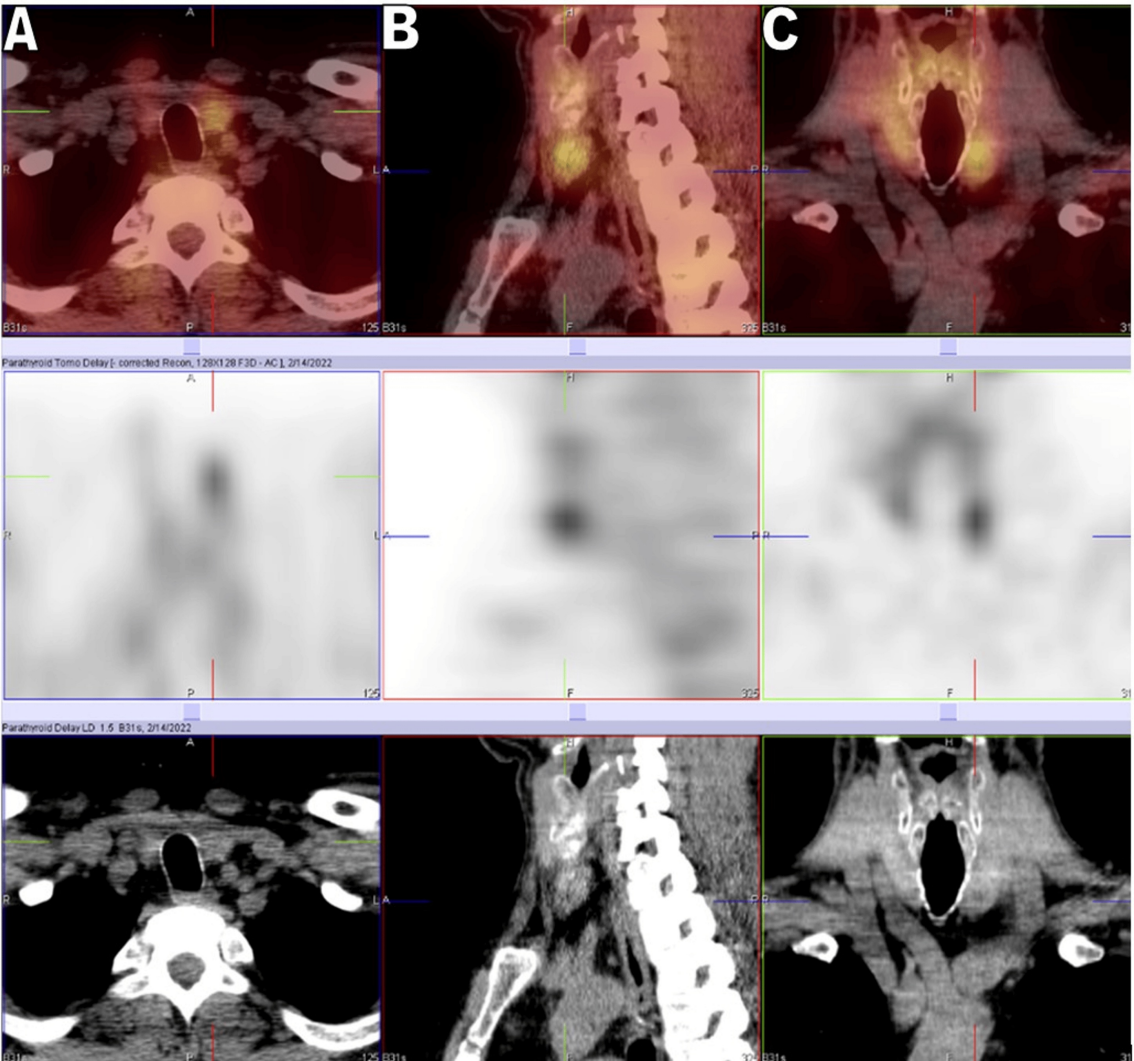
The SPECT/CT study following IV administration of 21 mCi Tc-99m labeled sestamibi. Images were acquired at 30 minutes and 2.5 hours. There is a small focal uptake at the inferior pole of the left lobe of the thyroid gland, which persists in the delayed phase, suspected to be a parathyroid adenoma. (A) Transverse view; (B) Sagittal view; (C) Coronal view SPECT: Single photon emission computed tomography

Neck ultrasound (US) found two thyroid nodules, one mm in the right thyroid lobe and another one measuring 11 mm in the left lobe. The patient was examined first by parathyroid single photon emission computed tomography (SPECT) methoxyisobutylisonitrile (MIBI) and then by thyroid scan with SPECT after IV administration of 21 mCi Tc-pertechnetate (Figure 1). The fine needle aspiration cytology (FNAC) revealed parathyroid cellular proliferation with high PTH levels in the blood but no evidence of thyroid follicular cells.

**Figure 2:**
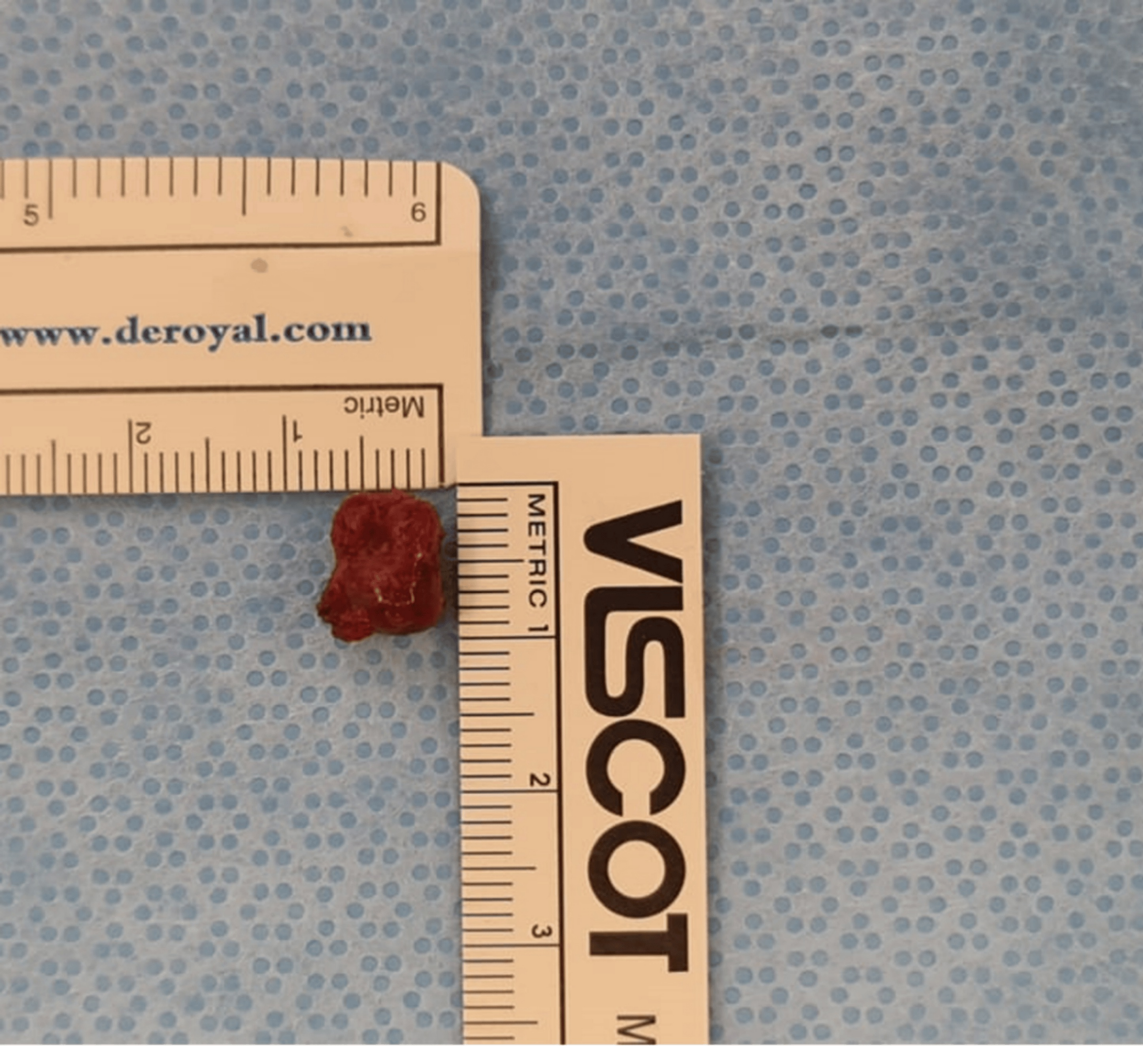
An abnormal parathyroid adenoma measuring 1 cm x 0.9 cm x 0.9 cm and weighing 0.35 g

The patient was admitted to the ENT surgical department to undergo adenoma excision by cervicotomy with laryngeal nerve monitoring. Under general anesthesia, a left cervicotomy was performed and the PTH levels were simultaneously requested. The thyroid gland was identified. The inferior pole of the left thyroid lobe had been dissected and elevated revealing one small brownish tissue resembling a parathyroid that was removed. Immediately after, PTH levels dropped down but still remained at a high level. The left lobe of the thyroid was digitally examined appreciating a nodule inside. On opening the lobe, a parathyroid tissue was found completely embedded within the thyroid tissue. The parathyroid adenoma was excised with the preservation of the thyroid tissues (Figure 2).

The patient recovered smoothly with no post-operative complications and was discharged on the second postoperative day after the removal of the drain. The experienced senior surgeon performed the procedure (AMP). The last results of laboratory examinations that was done one year after the surgery showed normal levels (Table 2). The nuclear medicine dual-energy X-ray absorptiometry (NM DEXA)-bone mineral density done preoperatively were within the expected range.

**Table 2:** The blood test results postoperatively PTH: Parathyroid hormone; Ca: Calcium; TSH: Thyroid-stimulating hormone

Blood work	Patient’s test results	Reference ranges
PTH	3.24 pmol/L	1.59-6.8 pmol/L
Serum Ca	2.34 mmol/L	2.1-2.5 mmol/L
TSH	0.88 mU/L	0.5-5.0 mU/L
FT4	16.2 pmol/L	12.0 - 22.0 pmol/L

**Figure 3:**
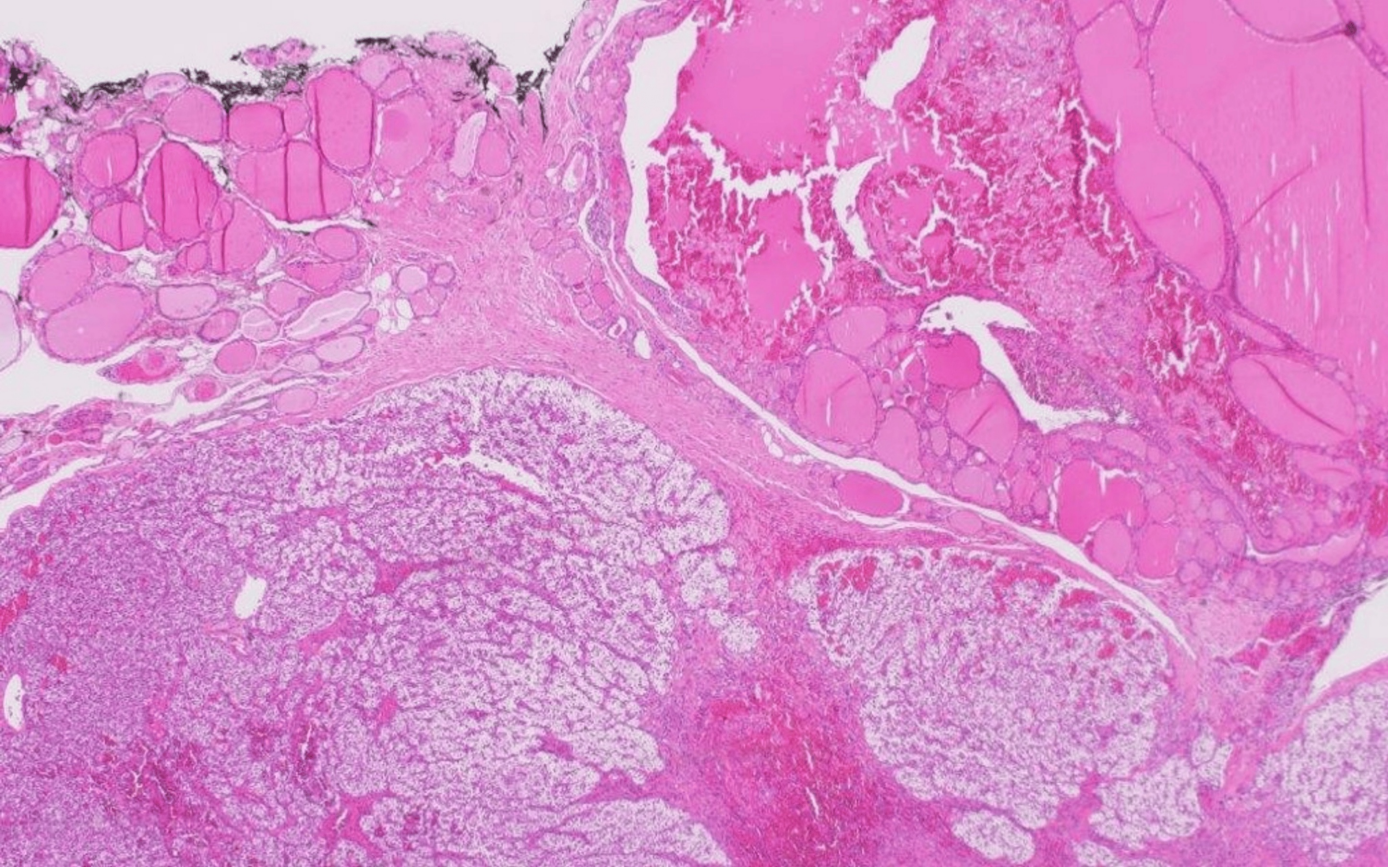
20-power magnification of H&E staining of our case showing parathyroid gland surrounded by thyroid tissue H&E: Hematoxylin and eosin

The histopathology examination revealed a well-circumscribed nodule with neoplastic cellular proliferation associated with decreased to absent stromal adipocytes with no evidence of atypia or lymphovascular invasion. The lesion was embedded in benign thyroidal tissue. There was no increase in mitotic activity (Figure 3). The first enlarged parathyroid removed was histologically unremarkable (left parathyroid tissue measuring 2 cm x 0.5 cm x 0.4 cm).

## Discussion

Literature suggests that parathyroid ectopia occurs in 4-20% of patients as a consequence of abnormal migration during embryogenesis [6]. It has been suggested that the superior parathyroid gland may become trapped within the thyroid as the lateral and medial lobes fuse, resulting in an intrathyroidal superior parathyroid gland, the inferior glands could presumably become trapped by the same mechanism and become intrathyroidal as well [7]. The vast majority occurs in the lower lateral quadrant and a small percentage near the recurrent nerve and superior pole.

Various imaging techniques, including cervical US, contrast-enhanced CT, and MIBI scintigram, are used for localizing parathyroid tumors. However, interpreting imaging findings for iT-PAs can be challenging. Typical US findings for iT-PA include an oval to near-spherical shape, a lack of fatty appearance, and the presence of feeding vessels, though these findings lack consistent diagnostic utility. CT imaging is limited by contrast enhancement from the background thyroid, making it difficult to confirm the tumor contrast pattern. SPECT/CT imaging has demonstrated effectiveness in the localization of parathyroid lesions, even in cases where slow washout, a feature common in thyroid nodules, is observed. Radio-guided surgery by injection of 10 mCi of TC-99m sestamibi and ex-vivo radionuclide detection by gamma probe has been suggested [5].

The use of US examination is an accurate method for identifying parathyroid adenomas in the cervical region, particularly when differentiating them from thyroid nodules and lymph nodes. US has a superior spatial resolution, is cost-effective, and is widely available [8]. However, due to limited acoustic windows, this method may not detect rare ectopic parathyroid glands (ETPGs) located in certain regions. In cases where ectopic parathyroid is suspected, scintigraphy, specifically Technetium 99-sestamibi scintigraphy, is recommended [9]. Single photon emission computed tomography/computed tomography (SPECT/CT) provides a combined functional and anatomical approach, enhancing the precise localization of ectopic parathyroid adenomas. Measuring PTH in thyroid fine-needle aspirates is also an effective way to diagnose intrathyroidal parathyroid pathology [10]. A definitive preoperative diagnosis of iT-PA remains challenging, and a combination of imaging techniques and cytology is recommended for accurate diagnosis.

In the presented case the US was unable to differentiate the adenoma from the thyroid tissue but the Tc-99m sestamibi was highly suggestive and the FNAC was able to demonstrate the presence of intrathyroid adenoma. In previous literature, blind ipsilateral thyroidectomy or subtotal thyroid lobectomy has been considered a potentially curative procedure for hyperparathyroidism when the mass is not easy to detect and previously it has been hypothesized to lie in an intrathyroidal location [7]. The efficiency of total lobectomy as a surgical approach for treating parathyroid adenomas has been a topic of debate, with some authors arguing that this approach may not be effective due to the relatively high occurrence of parathyroid adenomas in the contralateral lobe. Lobectomy/hemithyroidectomy is effective but with the disadvantage of increase risk of post operative hypothyroidism and could have also high rate of failure to remove the parathyroid adenoma [11].

Few cases of parathyroid adenoma enucleation are reported in literature. The major criticism is it can expose to have some remnants of parathyroid tissue increasing the possibility of adenoma recurrence [3,12]. The FNAC itself can damage and alters the parathyroid adenoma capsule giving capsular pseudoinvasion and tumor implantation out and not connected with the main one [2].

In our case, the parathyroid capsule was incomplete most probably related to the previous FNAC but the lesion has been removed embedded in thyroid tissue avoiding the possibility to leave in place/disseminate adenoma cells. Intraoperative PTH measurement in venous blood is highly recommended after the surgical removal to improve accuracy in identifying the ectopic location of parathyroid adenomas and have proof of its radical excision.

We have compiled and summarized previously reported cases of iT-PA, and 21 cases have been reported before our case (Table 3).

**Table 3:** Demographic, clinical, radiological, and result of treatment for 20 previously published cases of iT-PA PTH: Parathyroid hormone; iPTH: Intact parathyroid hormone; EMG: Electromyography; Ca: Calcium; TFT: Thyroid function test; SPECT: Single photon emission computed tomography; iT-PA: Intrathyroid parathyroid adenoma; ETPG: Ectopic parathyroid gland; MIBI: Methoxyisobutylisonitrile; PHPT: Primary hyperparathyroidism

Case	Author	Sex	Age (Yrs)	Clinical presentation	Lab findings	Initial imaging findings	Management	Outcome
1	Zhu et al. (2009) [13]	M	49	Weakness in the lower limbs of two years duration	Elevated Ca, elevated PTH, EMG showed myopathic features	CT of the neck revealed a parenchymatous tumor in the right lobe of the thyroid, scintigraphy showed a 'cold nodule'		The frozen section examination confirmed the diagnosis of an iT-PA
2	Michaud et al. (2015) [19]	F	52	Bone pain	Hypercalcemia, PTH level was elevated and the thyroid function was normal.	Left lobe of the thyroid gland had a weak echo, regular shape, and clear boundary	Left parathyroid adenoma resection, right partial thyroidectomy, and parathyroid exploration	The symptoms were relieved rapidly, and the serum free Ca level recovered
3	Kaushal et al. (2010) [14]	F	46	None	Hypercalcemia and elevated PTH levels	An abnormal 99mTc-MIBI uptake in an intrathyroidal region of the lower pole of the right lobe of the thyroid.	Right lobectomy	iT-PA, embedded in the thyroid tissue
4	Silaghi et al. (2011) [15]	F	48	Malaise, weight loss, generalized bone pain	Elevated serum levels of Ca and parathormone	4 cm nodular mass in the right thyroid lobe	Total right lobectomy	Intraoperatively, the adenoma was found in intrathyroidal location
5	Vilallonga et al. (2012) [16]	F	19	Muscle weakness, fatigue and a worsening of depressive symptoms	Life-threatening hypercalcaemia	47 × 22 mm nodule in the left thyroid lobe.	A hemithyroidectomy was performed	A clear intrathyroidal adenoma surrounded by thyroid tissue was observed
6	Cating-Cabral et al. (2012) [17]	F	44	A right-sided anterior neck mass	Primary hypothyroidism, Thyroid peroxidase antibody was elevated	A hypoechoic thyroid gland consistent with chronic lymphocytic thyroiditis with an enlarged cystic right thyroid lobe	Right thyroid lobectomy	A 5 cm parathyroid adenoma within the thyroid gland, surrounded by a pseudocapsule of thyroid tissue
7	Felix et al. (2015) [18]	M	48	Low-back pain	High serum Ca level and phosphate with iPTH	4 cm right-sided nodule in the region of the thyroid gland.	Localized right parathyroid adenoma removal	SPECT-CT confirmed a large right parathyroid adenoma
8	Sadacharan et al. (2015) [20]	F	42	None	Residual PHPT	Localization of parathyroid tumour on ultrasonography and sestamibi scintigraphy on the right side of the thyroid gland	A hemithyroidectomy	A parathyroid adenoma encircled by a rim of thyroid tissue confirming its intrathyroidal location
9	Doğan et al. (2015) [21]	F	69	Weakness, nausea, vomiting & depression	Serum Ca and PTH levels	US and parathyroid scintigraphy revealed hypoechoic nodules in right lobe, pieces of nodules in left lobe	Right parathyroidectomy and right total and left subtotal thyroidectomy	A parathyroid adenoma localized inside large thyroid nodules
10	Pirela et al. (2016) [22]	F	34	A nontender goiter	Abnormal TFTs	An intrathyroidal nodule consistent with a clear cell neoplasm of parathyroid origin	Right thyroid lobectomy and isthmusectomy	
11	Llorente et al. (2016) [23]	F	49	Bipolar disorder, in treatment with lithium for 20 years	Hypercalcaemia during routine lab work	Hypoechoic retrothyroid nodule adjacent to the inferior pole of the right thyroid lobe	Total thyroidectomy, the left inferior parathyroid gland could not be located	Parathyroid adenoma in the left thyroid lobe
12	Rutledge et al. (2016) [24]	F	21	Three-week history of an enlarging right sided neck mass	TFT normal. Corrected Ca was elevated with PTH level was over 20 times	A solid and cystic nodule in the lower pole of the right lobe of her thyroid	A right-sided parathyroidectomy, right thyroid lobectomy, and level VI neck dissection	An encapsulated multiloculated solid cystic mass, an atypical parathyroid adenoma
13	Kageyama et al. (2017) [25]	F	66	Treated for acute pancreatitis twice	Hypercalcemia	A soft tissue mass within the left lobe of the thyroid and scintigraphy parathyroid adenoma	Left hemithyroidectomy	The elevated Ca and iPTH were normalized. The patient has had no episodes of pancreatitis
14	Yang et al. (2019) [26]	M	53	Malignant multifocal thyroid nodules by US two years after kidney transplantation	Hypercalcaemia and persistent hyperparathyroidism	Coexistence of right iT-PAs, left cystic parathyroid nodular hyperplasia	Underwent bilateral neck exploration	The lesion was located in the right inferior thyroid and completely embedded within thyroid tissue
15	Cadena-Pineros et al. (2019) [27]	F	58	None	Decrease of Ca to 10.46 mg/d and reduction of iPTH	99mTc sestamibi (SPECT/CT), confirmed an inferior left intrathyroidal adenoma	Left hemithyroidectomy. revealed an iT-PA	Two years postoperative last surgery, serum Ca and iPTH had a normal levels
16	Chen et al. (2019) [28]	F	27	None	Thyroid function tests were normal	A hypoechoic nodule with multiple punctate microcalcifications within the right lobe	Right thyroidectomy, central compartment neck dissection, parathyroid exploration,	Auto-transplantation of normal ETPG
17	Takemoto et al. (2020) [6]	M	53	None	Laboratory examinations were compatible with hyperparathyroidism	An irregular round shape and heterogeneous hyperechogenicity located in inferior pole	Resection of the tumor under collar incision	An iT-PA was diagnosed by frozen section
18	Papanikolaou et al. (2020) [10]	F	43	Painless palpable hard mass in the lower pole of right thyroid lobe without cervical lymphadenopathy	Hypercalcemia with high levels of PTH and normal kidney function	An encapsulated, partially solid parathyroid adenoma, with a cystic component hemorrhagic areas	Thyroidectomy, central neck lymph node dissection and neck exploration	
19	Al-Yahri et al. (2020) [29]	F	61	Generalized bone ache, polyuria and right neck mass	High Ca (2.74 mmol/L), high iPTH (111 pg/mL)	Complex nodule within right thyroid lobe. Sestamibi scan suggested parathyroid adenoma	Right hemithyroidectomy	First reported case of HPTC and iT-PA co-occurring within the same thyroid lobe
20	Guneyli et al. (2022) [30]	F	53	Fatigue lasting for six weeks	Increased parathormone level	Hyperechoic parathyroid adenoma in the right lobe of thyroid and multifocal PTC in the left lobe of thyroid	A total thyroidectomy procedure was performed	Parathormone level decreased to 5.3 pg/mL one day after surgery

## Conclusions

iT-PAs pose a challenge in managing PHPT. Diagnostic imaging techniques, including US and scintigraphy, aid in identifying and localizing ETPGs without having complete certainty of their location which can be only hypothesized.

The case reported, superior parathyroid embedded in the thyroid lobe, and the literature review highlights the successful management of type III iT-PA preserving the thyroid tissue (enucleation technique). This results in less morbidity with less chance of having postoperative hypothyroidism. The preservation of the thyroid tissue and its function makes it possible to carry out a future contralateral lobectomy in case of emerging pathology avoiding any replacement therapy.

The management of type III iT-PAs without thyroidectomy provides a viable alternative for selected PHPT patients. Advanced diagnostic imaging and individualized surgical approaches contribute to successful outcomes. Standardized guidelines are necessary to optimize the diagnostic workup and the management of these adenomas to improve patient outcomes.
